# Technical note on introducing a digital workflow for newborns with craniofacial anomalies based on intraoral scans - part I: 3D printed and milled palatal stimulation plate for trisomy 21

**DOI:** 10.1186/s12903-020-1001-4

**Published:** 2020-01-23

**Authors:** Alexander B. Xepapadeas, Christina Weise, K. Frank, S. Spintzyk, C. F. Poets, C. Wiechers, J. Arand, B. Koos

**Affiliations:** 1 Department of Orthodontics, University Hospital Tuebingen, Osianderstr, 2-8, 72076 Tuebingen, Germany; 2 Section “Medical Materials Science & Technology”, University Hospital Tuebingen, Osianderstr, 2-8, 72076 Tuebingen, Germany; 3 Department of Neonatology, University Hospital Tübingen, Calwerstr, 7, 72076 Tuebingen, Germany

**Keywords:** Additive manufacturing (AM), Digital workflow, Vat-polymerization, Subtractive manufacturing (SM), Cleft lip and palate (CLP), Down’s syndrome (DS), Robin-sequence (RS), Computer-aided design/computer-aided manufacturing (CAD/CAM), Computer-aided impression (CAI), Poly-ether-ether-ketone (PEEK), Intraoral scanning (IOS), Trisomy 21 (TS21), Castillo Morales

## Abstract

**Background:**

Advanced digital workflows in orthodontics and dentistry often require a combination of different software solutions to create patient appliances, which may be a complex and time-consuming process. The main objective of this technical note is to discuss treatment of craniofacial anomalies using digital technologies. We present a fully digital, linear workflow for manufacturing palatal plates for infants with craniofacial anomalies based on intraoral scanning. Switching to intraoral scanning in infant care is advantageous as taking conventional impressions carries the risk of impression material aspiration and/or infections caused by material remaining in the oronasal cavity.

**Material and methods:**

The fully digital linear workflow presented in this technical note can be used to design and manufacture palatal plates for cleft palate patients as well as infants with functional disorders. We describe the workflow implemented in an infant with trisomy 21. The maxilla was registered using a digital scanner and a stimulation plate was created using dental CAD software and an individual impression tray module on a virtual model. Plates were manufactured using both additive and subtractive methods. Methacrylate based light curing resin and Poly-Ether-Ether-Ketone were the materials used.

**Results:**

The palatal area was successfully scanned to create a virtual model. The plates fitted well onto the palatal area. Manual post-processing was necessary to optimize a functional ridge along the vestibular fold and remove support structures from the additively manufactured plate as well as the milled plate produced from a blank. The additively manufactured plate fitted better than the milled one.

**Conclusion:**

Implementing a fully digital linear workflow into clinical routine for treatment of neonates and infants with craniofacial disorders is feasible. The software solution presented here is suitable for this purpose and does not require additional software for the design. This is the key advantage of this workflow, which makes digital treatment accessible to all clinicians who want to deal with digital technology. Whether additive or subtractive manufacturing is preferred depends on the appliance material of choice and influences the fit of the appliance.

## Background

Digital intraoral scans in dentistry and orthodontics are on the verge of replacing conventional impression techniques. They are currently used in different areas of dental medicine with encouraging results concerning reproducibility and accuracy [1]**.**

In the orthodontic field there is a certain patient clientele with syndromic conditions where it is crucial to begin treatment as early as possible after birth. For these patients, conventional alginate impressions can be life-threatening, whereas intraoral scanning followed by a digital workflow can considerably facilitate treatment [2]. The main objective of this technical note is to describe an easy to implement digital workflow for patients with craniofacial anomalies. Orthodontic appliances such as the Castillo Morales® stimulation plate, used for Trisomy 21 (TS21) patients, help to combat subsequent speech impairment, feeding problems or breathing difficulties [3].

When it comes to manufacturing such appliances, the conventional procedure starts with an alginate impression of the upper jaw using a standard or individualized tray [4]. This carries the risk of impression fragment aspiration, resulting in respiratory obstruction and acute cyanosis during the procedure. In cleft lip and palate (CLP) impression material may also remain inside the cleft and can cause inflammation [4, 5]. In some cases, immediate intervention is necessary to ensure respiration and oxygen supply and resolve cyanosis [5, 6]. As a result, a team of neonatologists, orthodontists and neonatal nurses must be present when an impression is taken. In severe cases, children are intubated for impression taking which is a delicate procedure due to their general state of health. Thus, there is a need to adapt the workflow in order to reduce the overall risk for the patient.

In newborns and infants with craniofacial anomalies intraoral scanning (IOS) is deemed an adequate replacement for conventional impressions [7–9]. The fully digital linear workflow presented here offers a solution for plate design that is easy to implement and feasible for every clinician and dental technician. The newly developed workflow is clinically established and has been applied in over 50 cases for different syndromic anomalies, e.g. CLP or TS21, over the course of 6 months at Tuebingen University Hospital. Introducing IOS into daily clinical routine in the area of cleft care is a step towards faster, more reproducible and, importantly, less dangerous treatment concepts. From this point of view, the concept is transferable to the orthodontic treatment of neonates with a variety of diseases.

Different manufacturing methods and materials are emerging and open up new horizons with respect to digital workflows. Besides additive manufacturing (AM), milling is the technology which has generally substituted the traditional processing of precious metal casting alloys in dentistry where ceramics and polymers are milled as well [10]. In terms of AM palatal plates, Class IIa medically approved splint materials may be substitutes for conventional cold polymerizing plastics. From a flexural strength point of view, splint materials have proven comparable to conventional materials [11]. Milling, on the other hand, is an attractive method because materials are produced under highly controlled conditions, although the final result may still be affected by operating conditions [10]. Especially in orthodontics and dentistry, where biocompatibility and high mechanical resistance are indispensable**,** Poly-Ether-Ether-Ketone (PEEK) has great potential. PEEK is considered a high-performance thermoplastic polymer [12].

To prove its value, a fully digital linear workflow is presented below as implemented in a clinical case of an infant with Trisomy 21 (TS21). For comparison, the case involves both a subtractive and an additively manufactured stimulation plate following the Castillo Morales® treatment concept. To give further insight into the clinical case presented here as an example, a short summary of the clinical background is given in the following section.

TS21 is a genetic disorder associated with an incidence of 1 per 700 live births and significant mental retardation [3]. From an orthodontic point of view, it is associated with a generalized hypotonic orofacial musculature. The tongue is positioned extraorally and rests on the lower lip, which affects the physiological development of language and dentition. Mouth breathing and increased salivation lead to higher plaque accumulation and increased caries risk. Furthermore, there is skeletal maldevelopment into an Angle class III anomaly, sensomotoric dysfunction with a habitually open mouth, pseudomacroglossia and a high palate [13–16]. Our treatment concept involves the use of an orthodontic palatal stimulation plate combined with speech therapy based on the concept of Castillo Morales® [17]. It considers posture and mobility depending on muscular activity, especially in the facial area [16, 18, 19]. For cranioventral orientation of the tongue and activation of the upper lip muscles, combining the Castillo Morales® treatment concept with a stimulation plate has proven useful [20].

## Material and methods

### Intraoral scan

**Fig. 1:**
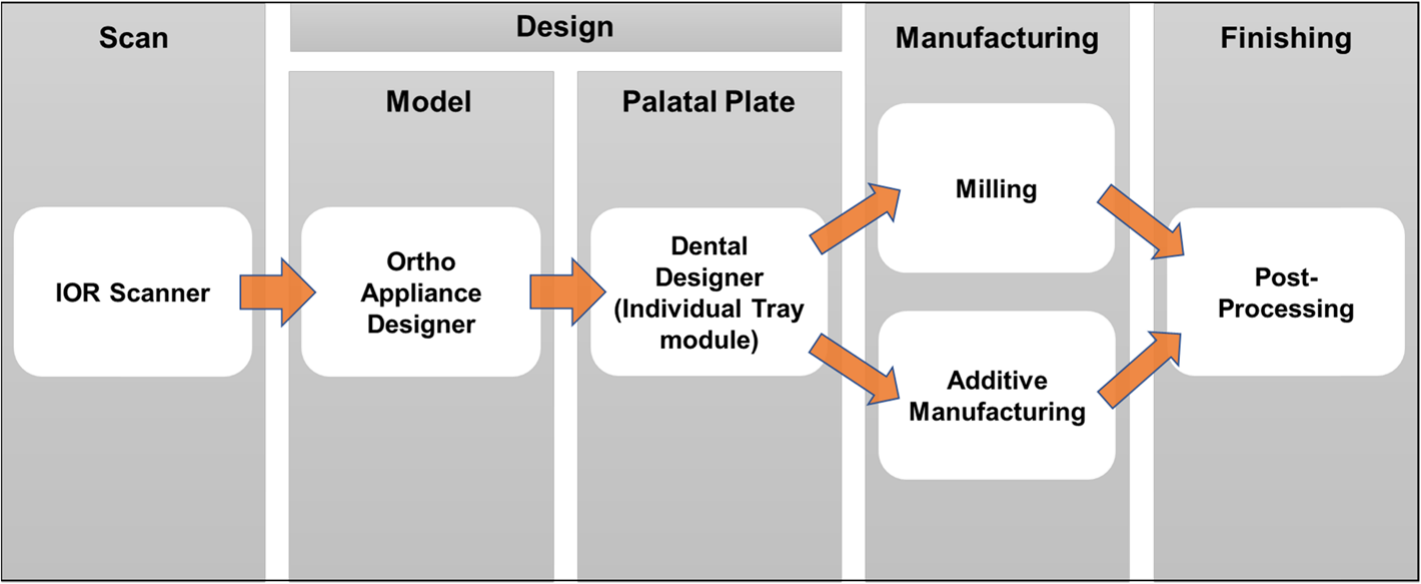
The digital workflow for manufacturing an orthodontic palatal plate

The digital workflow follows the sequence shown in Fig. 1. A digital impression has to be taken prior to manufacture of an orthodontic palatal plate. The upper jaw is registered by the Trios Scanning Software using the TRIOS3 intraoral scanner (3Shape, Copenhagen, Denmark). The acquisition software automatically registers scan duration. After acquisition of the raw scan, the “post-processing tool” calculates the surface of the scan. After saving, the file is sent to the 3Shape Ortho Appliance designer through 3Shape Direct Connect.

During the scan, the orthodontist is careful to include all areas that could influence the fit of the plate. The most important structures include the maxillary tuberosity, the vestibule and the labial frenulum. The scanner needs a defined reference point at which the scan is always started. This is the incisive papilla. From there, the alveolar ridge is scanned to the right, together with the tuberosity and the vestibule. If the scanner loses its scanning position, the incisive papilla or the last successfully scanned areas are taken again as a starting point. Once the right alveolar ridge, tuberosity and vestibule have been scanned, the left part of the jaw is scanned, starting at the incisive papilla. Finally, the vestibule in the anterior region of the maxilla is scanned by tilting the scanner in its vertical axis. This includes the labial and buccal frenula.

### Design of a Working Model

**Fig. 2:**
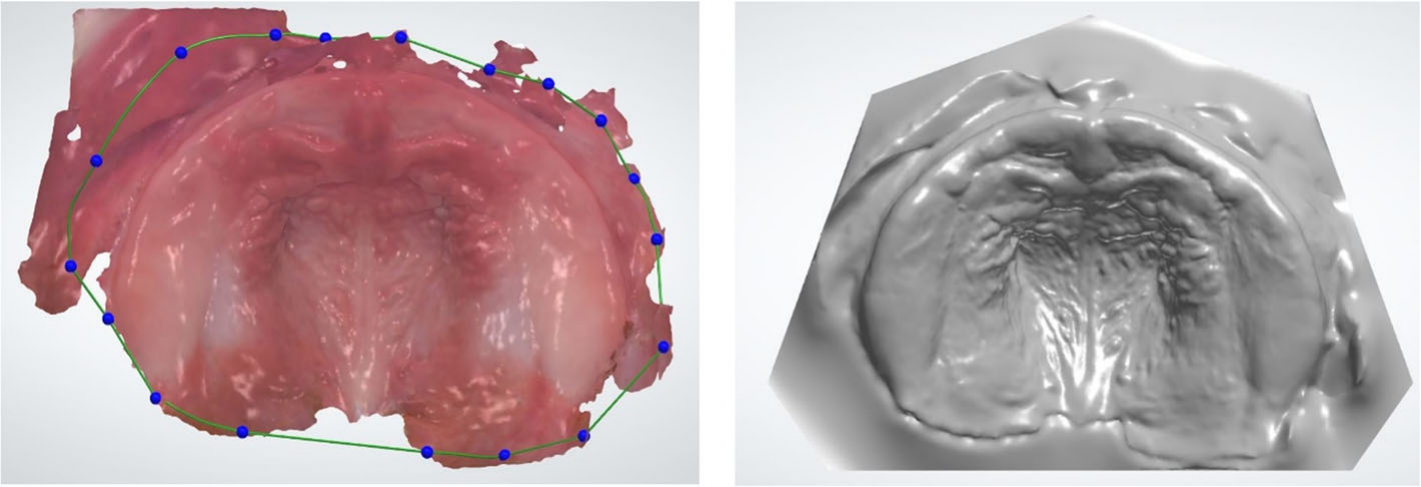
Intraoral Scan with drawn contour of the scan (left), finished model (right)

After importing the scan data into the 3Shape Ortho Appliance designer, the digital working model is created using the Model Creator module (3Shape) which is started directly from the appliance designer. First, the scan is oriented in the occlusal and sagittal planes. Second, the spline function is used to define the outer contour of the scan, which will be projected onto the model base later. Next, the scan must be positioned in the model base. This step determines the vertical and horizontal dimensions of the orthodontic model. The model is calculated by the software and can be refined in free form in a final step. This tool is used to remove undercuts or surface irregularities which are created by registration errors or missing surface information. In the case of CLP, the cleft can be blocked out virtually. To ensure that no important anatomical structures are accidently removed, the texture of the original scan can be faded in, which makes the identification of scan errors easier. Now the model is exported as a Standard Tessellation Language (STL) file and the appliance designer program is closed. If desired, the dental model can now be manufactured using AM technology. In Fig. 2, the contour (left) and the final model (right) are displayed.

### Design of a palatal plate

To produce an appliance, a new patient file is created in the Dental Designer software. The maxilla is chosen and the design mode “individual impression tray” is selected. After saving, the previously designed working model is imported as a “scanned model” and the design process can begin.

**Fig. 3:**
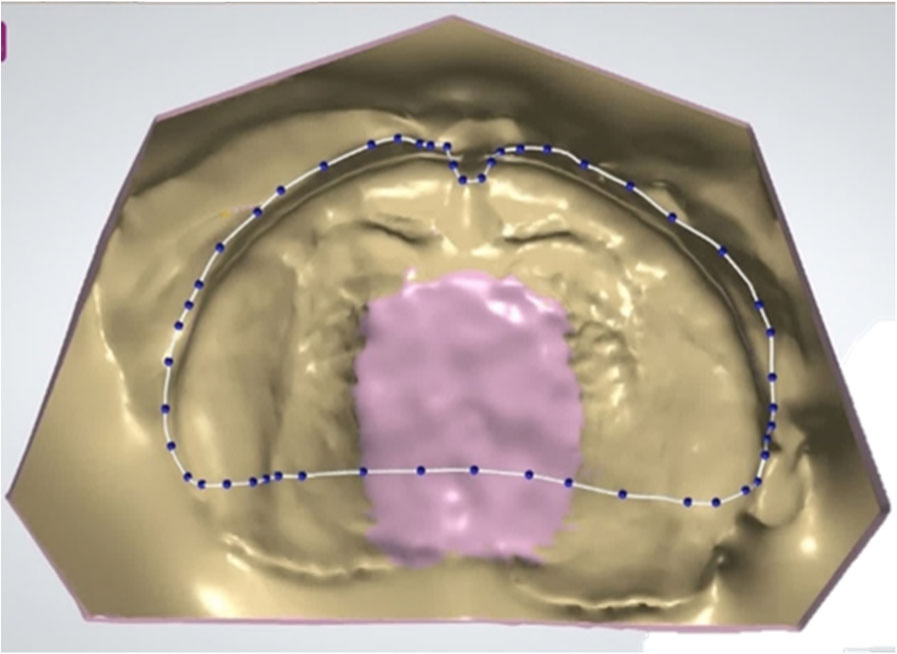
Contour of the palatal plate and blocked out high-palate on the virtual model, created by using the spline function

The insertion angle is set so large undercuts can be blocked out automatically. In addition, virtual wax can be applied manually in free form, e.g. if the palatal area is too deep to form a physiological anatomy on the plate. This is especially helpful in CLP patients. Once the model is prepared, the desired dimensions of the stimulation plate are drawn onto the model using the spline function. For the contour, it is important to spare the areas around the labial and buccal frenula. Furthermore, the maxillary tuberosity must be encased enough to guarantee a good fit without disturbing mandibular mobility and thereby injuring the patient. The posterior end is set along the vibrating line (Fig. 3).

Next, a plate is automatically created. Some user specific parameters have to be set to calculate the base. A base thickness of 2 mm, room for impression of 0 mm and a hollow of 0 mm are selected and the plate is automatically created directly on the palatal area. This results in an uneven surface, which can now be smoothed and sculpted in free form. A thickness of 1.5 mm will be preferable for very small patients. The technician must be careful to maintain the minimum material thickness in accordance with the material’s parameters when molding the plate in free form.

**Fig. 4:**
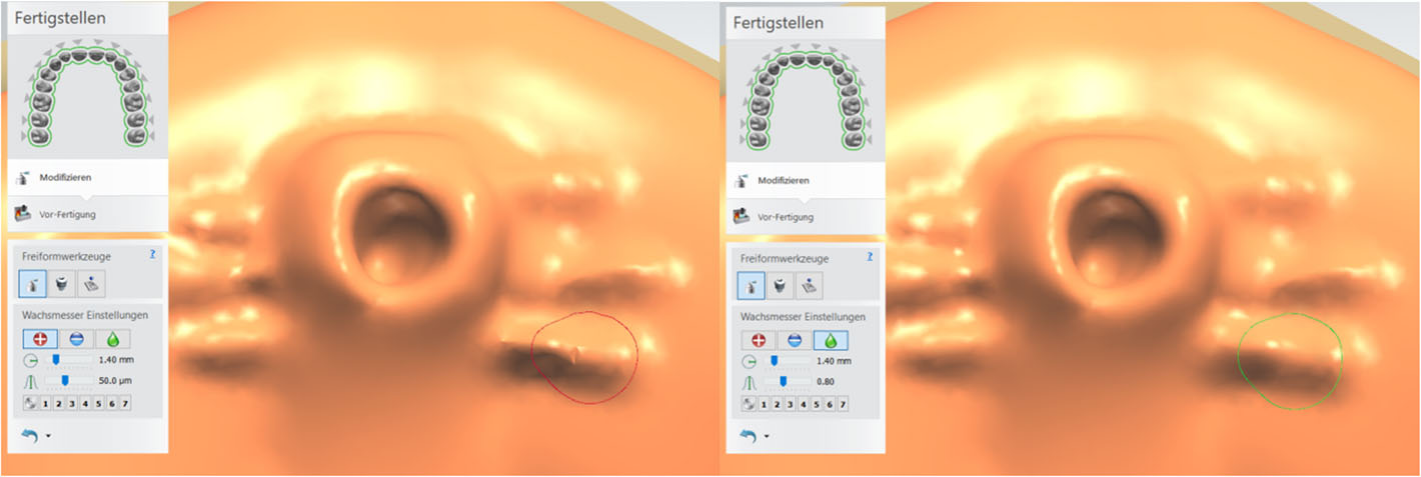
Design of the stimulation element in freeform (left). Smoothening tool to remove sharp edges (right)

**Fig. 5:**
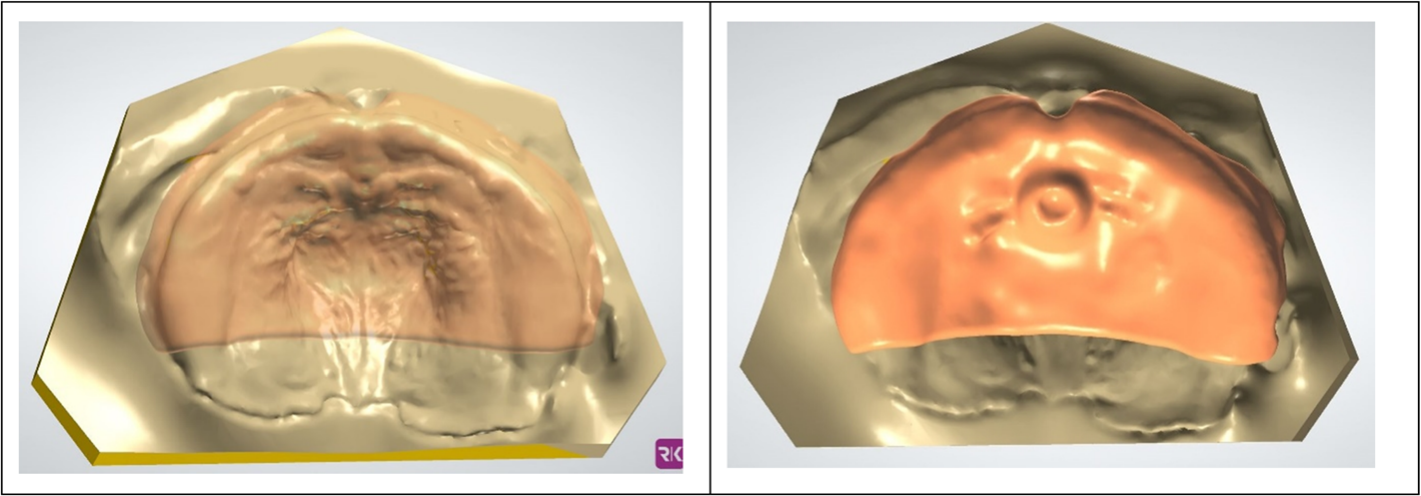
Palatal plates containing stimulation elements. Faded out (left) with stimulation element and palatal folds (right)

The alveolar ridge is brought to an even level to increase patient comfort. Additionally, sharp edges on the basal area are removed by using the smoothing tool or by removing material. The base plate is now complete, and a stimulation element can be added if necessary by using the free form tool (Fig. 4). To position the stimulation element in the intended area the plate is slightly faded out to the point where the underlying structures of the palate become visible. This enables exact positioning of the stimulation element onto the incisive papilla (Fig. 5). To increase stimulation and create a natural feeling in the palatal area, palatine rugal folds are created on the plate modeled on the original anatomical features, visible through the faded-out plate. The plate is exported as an STL-file for manufacturing.

### Additive manufacturing

For AM preparation, the STL-file for the stimulation plate is imported into Netfabb Premium 2018 (Autodesk, San Rafael, CA, US)., The building platform of the Solflex 170 (Way2 Production, Vienna, Austria) is displayed to virtually position the parts. The plate is positioned upright with its apical end towards the building platform. Additionally, it is slightly tilted so that no supports are needed on its basal side. Printing Supports are automatically generated with the support Script VOCO Splint V 1.0. For printing, layer height is set to 50 μm and the Material VPrint Splint (VOCO, Cuxhaven, Germany) selected. Now, the print job is calculated and transferred to the machine. The Direct-Light projection (DLP) printer Solflex 170 offers different manufacturing modes, whereby printing speed can be adjusted. The printing mode “rapid” is chosen to create the appliance as fast as possible.

After manufacturing, the parts are left to drip off in the printer for 10 min. The plate is removed and immersed in Isopropanol (IPA) in an ultrasonic bath for 3 min. The IPA is removed with compressed air and the plate taken off the platform. Now the plate is immersed in IPA a second time for 2 min and dried again with pressurized air. All support structures are removed using a wire cutter. The plate is UV-post-cured twice with 2000 flashes in an Otoflash G171 (NK-Optiks, Baierbrunn, Germany).

### Subtractive manufacturing

In the case presented here the same STL was used for subtractive manufacturing to permit direct comparison. The appliance was manufactured using an Organical Multi 5 X milling machine (Organical CAD/CAM GmbH, Berlin, Germany) to test the applicability of milled PEEK as a potential material for the manufacturing of palatal plates.

### Manual post-processing

The areas where supports were placed as well as the functional ridges are smoothed using a fine gypsum cutter to avoid prying of the appliance. The functional ridge must be manually adapted because the software creates rather thick edges. The surface is post-processed using sandpaper (150 grid) and mechanical polishing on the lingual side only; first with a muslin buffing wheel and powdered pumice, then using polishing paste.

### Case presentation

**Fig. 6:**
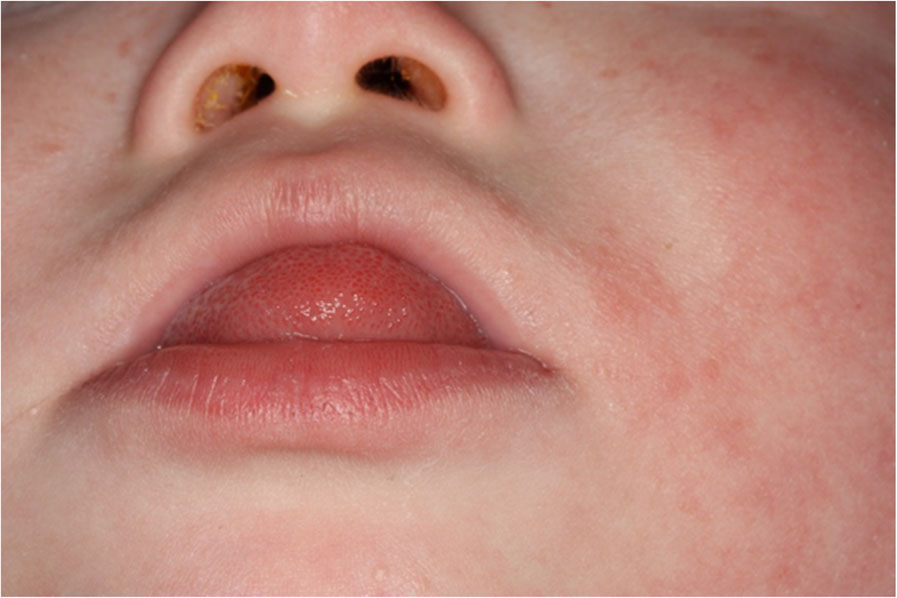
Typical tonge position in patients with trisomy 21

The patient with TS21 presented here as a clinical example had the hypotonic, perioral musculature and macroglossia typical of this syndrome (Fig. 6). Upon first admission at an age of 3 months, a sleep study was conducted to evaluate the degree of airway obstruction. This showed a mixed-obstructive apnea index (MOAI) of 0.6/h (normal). The patient was subsequently treated with a Castillo Morales® stimulation plate, manufactured from cold-polymerizing methacrylate Orthocryl (Dentaurum, Ispringen, D) to stimulate the hypotonic musculature and improve tongue position and tonus. A second polysomnography was performed after 10 months which resulted in a MOAI of 0.1/h. As the first stimulation plate had grown too small, a new plate was manufactured using the digital workflow.

**Fig. 7:**
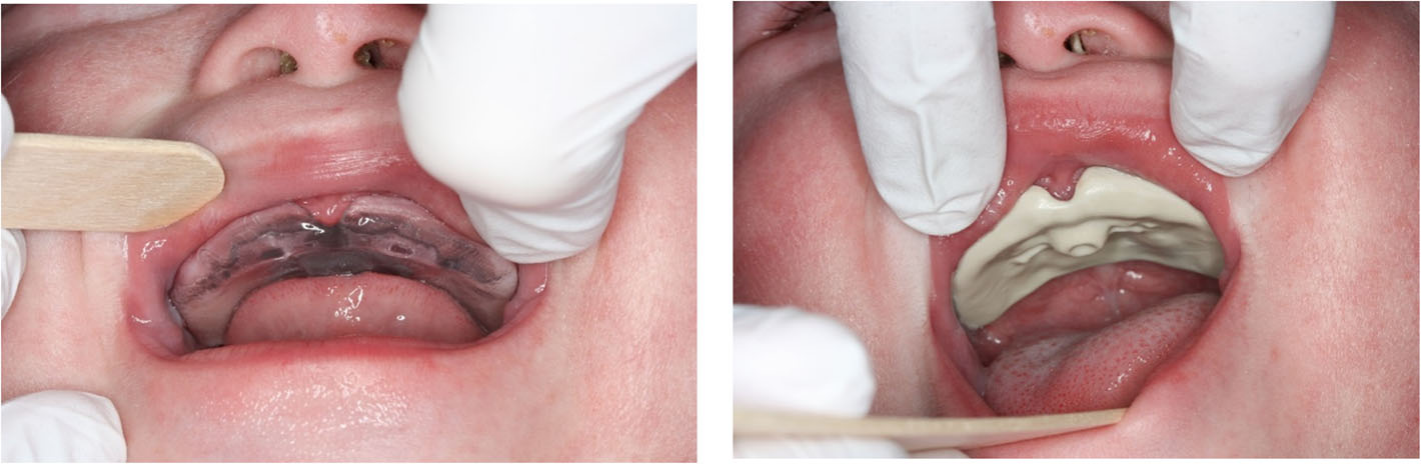
Additive manufactured (left ) and milled (right) palatal stimulation plates

In Fig. 7 the AM and milled plates, created with the digital workflow, are displayed. Both plates were inserted in the presence of the infant’s parents to compare their fit. The correct intra-oral position was controlled clinically. Chairside adaption of plates was not necessary. The additively manufactured plate had a better fit than the milled one. The milled plate became loose after a short period of time during the clinical evaluation, whereas the AM one stayed in place longer without adhesive cream. Immediately after introducing the plate, the patient reacted to the stimulation element. The AM plate was chosen for further treatment because it fitted better.

## Discussion

Our case illustrates a completely digital linear workflow to produce orthodontic palatal plates. Scan duration for this patient was 4:15 mins. Scanning was complicated by the hypotonic musculature and enlarged tongue in addition to uncontrolled salivation. Here, relevant areas can be cleaned before scanning. In general, scanning-duration should decrease as scanning strategy and experience improve [21].

For model and plate design, it is most important to record the tuberosity and the vestibular fold. Furthermore, an exact representation of the distal area is necessary to guarantee a good plate fit. This was achieved successfully using the Trios 3 intraoral scanner. If the plate extends too far into the dorsal area, stability and fit may be compromised and pressure marks may occur in the area of the soft palate. The vestibular fold is important for plate design as it enables the operative to create a plate with maximum edge-length for best retention. However, the technician must be careful not to cover any mobile anatomical areas of the vestibule such as the labial and buccal regions, to ensure retention of the appliance and avoid pressure marks and irritations. Good communication between the technician and the clinician performing the scan is essential to guaranteeing ideal plate design. In our experience intraoral scans are precise enough to manufacture palatal plates with sufficient clinical fit. The manufacturing method and the choice of material influence the fit of the plates. This needs to be evaluated in future studies.

Chalmers et al. already postulated 4 years ago that conventional impressions should be replaced by intraoral scanning in CLP patients [7]. The above procedure can be seen as an advancement to other workflows which involve AM palatal plates, where a digitized plaster model was used as a basis and non-dental CAD software was used for plate creation [22]. The authors postulated that in order to use intraoral scanning, improved soft tissue detection is necessary. Krey et al. were able to successfully scan jaws for a complete digital workflow with a different scanner. They saw limitations in the resolution of the scanner, which makes it difficult to reliably detect the tuberous areas and the cleft. Additionally, they called for a miniaturization of the scanner head as an improvement [9]. Although we agree that the field depth is an important hardware factor, we believe that scanning strategy will prevail over scanner-head size. A reduced scanner head size would also minimize the field of view. This could make it more difficult for the scanning software to “stitch” the images together and the scanner could lose its orientation more easily. In a comparison of seven different IOS, the TRIOS 3 was proven to provide the best combination of scanning speed, trueness and precision [1]. This may also have positively influenced our results concerning the fit of the plates.

Due to the accuracy of the design process and the AM, manual finishing of the printed plates is hardly necessary. Nevertheless, post-processing after printing has a great influence on the material’s stability. It is crucial to strictly follow the manufacturer’s instructions concerning washing time in IPA and the choice of light-curing device [11].

PEEK as a material is prone to temperature deformations during manufacturing which may influence the fit of appliances [23]. Decreasing the amount of retentive structures on the plate by adapting the CAD-design might decrease the heat-energy produced during manufacturing [24]. If a plate detaches a few seconds after insertion, this might be attributed to its greater weight. To prevent the plate from detaching, the retentive force could be increased by designing the plate to match the anatomical structures as closely as possible. Additionally, an unmodified rough inner surface improves retention. The PEEK material used in this case had a very smooth surface and therefore did not offer much retention for the adhesive cream. Generally, milling parameters such as the feed rate, milling speed and depth of cut have a great influence on surface quality and can influence roughness [25]. Lowering the feed rate could thus improve fit although possibly at the expense of time efficiency in daily clinical routine. Concerning CAD design, it should still be possible to improve the fit of the milled components by performing a cutter-radius correction in the CAD software.

Disadvantages of a digital approach include that it is not yet possible to incorporate elements like screws, which enable the plate to “grow” with the patient or induce a transverse movement of the alveolar segments in cleft palate patients. Recently, however, a correction factor for palatal plate production due to age-related growth was introduced, which would make a screw obsolete [26]. As expansion of the jaw mostly occurs in the transverse plane during the first months of life, pressure marks in the lateral areas of the tuberosity indicate that the plate has become too small. The conventional procedure involves removing material in this area, which is only possible up to a certain point, then manufacturing a new plate. One solution would be to include the above-mentioned growth factor which can be incorporated into the digital workflow.

## Conclusion

Digital intraoral impressions are a safe alternative to conventional impressions for infants with craniofacial anomalies and enable the production of clinically relevant appliances. The low-risk digital workflow described here is clinically established in our center and has the potential to completely replace the conventional workflow in other centers as well. Besides improving scanning technology, choice of material will be key to further developing this workflow. Whether additive or subtractive manufacturing is preferred depends on the material of choice and has an influence on the fit of the appliance. In summary, AM is the preferred method with this digital workflow based on our experience.

## Data Availability

All data and materials are accessible on a local server of the Department of Orthodontics of the University Hospital Tuebingen Germany.

## Abbreviations

AM: Additive Manufacturing
CAD/CAM: Computer-Aided Design/Computer-Aided Manufacturing
CLP: Cleft lip and palate
IOS: Intraoral Scanner
MOT: Manual Orofacial Therapy
OPPT: Orthodontic palatal plate therapy
POT: Presurgical Orthognathic Treatment
RS: Robin Sequence
STL: Standard Tessellation Language
TPP: Tübingen Palatal Plate
TS21: Trisomy 21/ Down’s Syndrome
UAO: Upper Airway Obstruction
